# Automatic diagnosis of retention pseudocyst in the maxillary sinus on panoramic radiographs using a convolutional neural network algorithm

**DOI:** 10.1038/s41598-023-29890-5

**Published:** 2023-02-15

**Authors:** Eun-Gyu Ha, Kug Jin Jeon, Hanseung Choi, Chena Lee, Yoon Joo Choi, Sang-Sun Han

**Affiliations:** Department of Oral and Maxillofacial Radiology, Yonsei University College of Dentistry, 50-1 Yonsei-Ro Seodaemun-Gu, Seoul, 03722 Korea

**Keywords:** Anatomy, Diseases

## Abstract

The evaluation of the maxillary sinus is very important in dental practice such as tooth extraction and implantation because of its proximity to the teeth, but it is not easy to evaluate because of the overlapping structures such as the maxilla and the zygoma on panoramic radiographs. When doom-shaped retention pseudocysts are observed in sinus on panoramic radiographs, they are often misdiagnosed as cysts or tumors, and additional computed tomography is performed, resulting in unnecessary radiation exposure and cost. The purpose of this study was to develop a deep learning model that automatically classifies retention pseudocysts in the maxillary sinuses on panoramic radiographs. A total of 426 maxillary sinuses from panoramic radiographs of 213 patients were included in this study. These maxillary sinuses included 86 sinuses with retention pseudocysts, 261 healthy sinuses, and 79 sinuses with cysts or tumors. An EfficientDet model first introduced by Tan for detecting and classifying the maxillary sinuses was developed. The developed model was trained for 200 times on the training and validation datasets (342 sinuses), and the model performance was evaluated in terms of accuracy, sensitivity, and specificity on the test dataset (21 retention pseudocysts, 43 healthy sinuses, and 20 cysts or tumors). The accuracy of the model for classifying retention pseudocysts was 81%, and the model also showed higher accuracy for classifying healthy sinuses and cysts or tumors (98% and 90%, respectively). One of the 21 retention pseudocysts in the test dataset was misdiagnosed as a cyst or tumor. The proposed model for automatically classifying retention pseudocysts in the maxillary sinuses on panoramic radiographs showed excellent diagnostic performance. This model could help clinicians automatically diagnose the maxillary sinuses on panoramic radiographs.

## Introduction

The maxillary sinus is the largest air-filled space around the nose and occupies most of the maxillary bone^1^. Because it is close to the teeth, evaluation of the maxillary sinus plays an important role in dentistry^2^. Retention pseudocysts (mucous retention cysts or pseudocysts) in the maxillary sinus occur through multiple related conditions leading to the development of cyst-like lesions that are not lined by epithelium^3^. These lesions present as well-defined, dome-shaped, hemispherical or circular radiopacities on panoramic radiographs^4^ and are often confused with cysts or tumors. Retention pseudocysts resolve spontaneously and do not require treatment^5^, but cysts or tumors require surgery; therefore, the differential diagnosis is very important.

The primary evaluation of the maxillary sinus is conducted using panoramic images in dental clinics. Since panoramic radiographs are two-dimensional images, it is sometimes difficult to accurately evaluate the maxillary sinus due to the overlapping of several structures, irregular magnification, and distortion^6–8^. Retention pseudocysts may be mistaken for cysts and tumors, and these errors are particularly frequent among inexperienced dentists or those who need to diagnose many images in short period of time. The accurate diagnosis of retention pseudocysts is important because misdiagnosing pseudocysts as cysts or tumors could increase patients’ radiation exposure and cost due to the need for additional imaging, such as computed tomography (CT).

In the dental field, many artificial intelligence (AI)-based studies using convolutional neural networks (CNNs) have been conducted on panoramic radiographs^9–11^, and several studies about maxillary sinuses have been reported^12–14^. Kuwana et al.^12^ developed the DetectNet model, which classifies sinuses as healthy, inflamed, or containing a cyst or tumor, but that study included pseudocysts within the cyst group. Other studies^13,14^ have proposed models to distinguish between healthy and inflamed sinuses. However, no studies have yet differentiated retention pseudocysts.

Therefore, we hypothesized that the maxillary sinus could be automatically diagnosed into a novel categorization (healthy, retention pseudocyst, and cyst or tumor) on panoramic radiographs using CNNs model. The main purpose of this study was to develop a deep learning model to automatically classify retention pseudocysts in the maxillary sinuses on panoramic radiographs using the EfficientDet algorithm.

## Materials and method

### Subjects

This study was approved by the Institutional Review Board of Yonsei University Dental Hospital (No. 2-2022-0020) and was performed in accordance with ethical regulations and guidelines. The requirement for informed consent was waived by the Institutional Review Board of Yonsei University Dental Hospital due to the retrospective nature of the study, and all data in this study were used after anonymization.

In total, 213 patients who visited Yonsei University Dental Hospital from December 2016 to December 2021 and underwent both panoramic radiography and CT or cone-beam computed tomography (CBCT) were included in this study. The 426 maxillary sinuses were divided into the following three groups; healthy (261 sinuses), retention pseudocyst (86 sinuses), and cyst or tumor (79 sinuses). Healthy maxillary sinuses were defined as those with mucosal thickening less than 4 mm through CT or CBCT in the absence of clinical symptoms, as reported by Murata et al.^13^. The retention pseudocyst group consisted of cases presenting with dome-shaped soft tissue on CT or CBCT, and the cyst or tumor group consisted of cases containing a radicular cyst, dentigerous cyst, odontogenic keratocyst, postoperative maxillary cyst, or ameloblastoma. All cyst and tumor cases were histopathologically diagnosed after surgery.

### Preparation of imaging datasets

The panoramic radiographs were taken using two different types of equipment: RAYSCAN Alpha (Ray Co., Ltd., Hwaseong-si, Korea) and PaX-i3D Green (Vatech Co., Ltd., Hwaseong-si, Korea). All panoramic images with a size of 2993 (width) $\times$ 1500 (height) pixels were exported in bitmap format. The three different groups (healthy, retention pseudocyst, and cyst or tumor) were categorized as classes 0, 1, and 2. The distribution of maxillary sinuses in the training, validation, and test datasets in the proposed model is presented in detail in Table 1.

**Table 1:** Number of maxillary sinuses in the training, validation, and test datasets.

Group	Training	Validation	Test	Total
Healthy (class 0)	197	21	43	261
Retention pseudocyst (class 1)	60	5	21	86
Cyst or tumor (class 2)	54	5	20	79

The training and validation datasets were augmented using various methods, such as rotation, scaling, cropping, shifting, and changing brightness, to prevent overfitting due to the limited datasets^15^. As a result, the training and validation datasets were increased five-fold (one original and four augmented images of each panoramic image).

### Development and evaluation of the model

**Figure 1:**
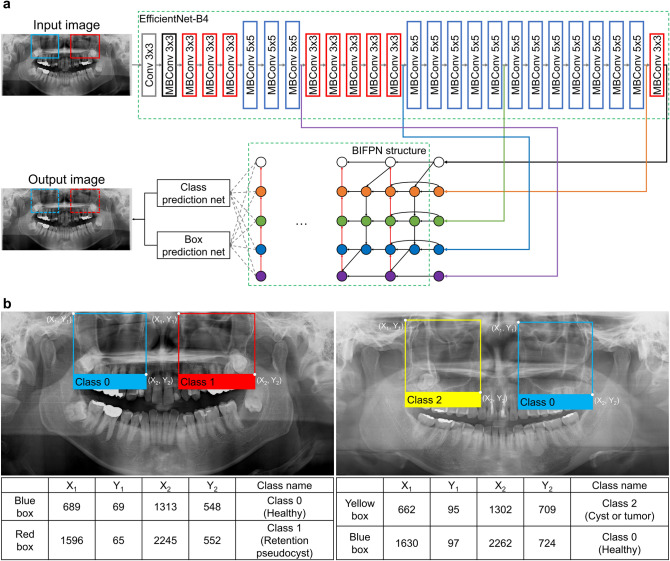
Model architecture and annotation. Overall architecture of the proposed model from EfficientDet-D4 (**a**). Conv, convolutional network; MBConv, mobile inverted bottleneck convolution block; BIFPN, bi-feature pyramid network. Examples of annotated panoramic radiographs and annotation information (**b**). The blue boxes indicate class 0 (healthy), the red box indicates class 1 (retention pseudocyst), and the yellow box indicates class 2 (cyst or tumor). The information was composed of the location, represented as the upper left (X_1_, Y_1_) and the lower right (X_2_, Y_2_), and each class name.

We developed a model based on the EfficientDet algorithm for the automatic classification of the maxillary sinus on panoramic radiographs. EfficientDet, a CNN algorithm for object detection, was first proposed by Tan et al.^16^ and it contains eight model structures (EfficientDet-D0–EfficientDet-D7). The EfficientDet algorithm employs the EfficientNet algorithm (EfficientNet-B0–EfficientNet-B7) as the backbone network and the Huber loss and focal loss as loss functions. A bi-feature pyramid network structure, which was first proposed in the EfficientDet algorithm, enables training by finding significant features through diverse fusion scales while varying the weights according to resolution. The EfficientDet algorithm showed good performance in medical images^17–19^, but few studies^20^ using this algorithm have been conducted in the dental field. In this study, we developed a model based on the EfficientDet-D4 algorithm^16^ to identify maxillary sinuses with their classes, considering the availability of limited computing resources (Fig. 1a).

The model needed information on the training and validation datasets, such as the location and class name of the ground truth. An oral radiologist with over 20 years of experience annotated the rectangular region of interest, including the maxillary sinuses, with the corresponding class name (class 0, 1 and 2) using the graphical image annotation tool LabelImg (version 1.8.4, available at https://github.com/tzutalin/labelImg). From the annotations, the coordinate values of the upper left (X_1_, Y_1_) and lower right (X_2_, Y_2_) and classes of the maxillary sinuses were extracted for training and validation datasets. The blue, red, and yellow boxes represent the annotated sinuses of class 0 (healthy), class 1 (retention pseudocyst), and class 2 (cyst or tumor), respectively (Fig. 1b). The extracted information, along with the input images, was used in the model training process.

The panoramic radiographs were resized to 512 (width) × 256 (height) pixels, and the model was trained 200 times with our training and validation datasets using pre-trained weights from the COCO dataset as initial weights. When the trained model detected and classified a sinus, it output an image marked with a box in a different color for each class (healthy: blue, retention pseudocyst: red, cyst or tumor: yellow) in the detected area. If no sinus was detected, it provided the input image without a box. The trained model was deemed to have correctly predicted the sinuses only if the intersection over union (IoU) value between the detected area and ground truth was 0.5 or higher^21^. After training the model 200 times, the performance of the trained model was validated according to whether each class was accurately detected and classified on test dataset using the following formula.

$$\mathrm{Detection}\,\mathrm{accuracy}=\frac{\mathrm{Number}\,\mathrm{of}\,\mathrm{correctly}\,\mathrm{detected}\, \mathrm{and}\,\mathrm{classified}\,\mathrm{maxillary}\,\mathrm{sinuses}\,\mathrm{in}\,\mathrm{each}\,\mathrm{class}}{\mathrm{Total}\,\mathrm{number}\,\mathrm{of}\,\mathrm{maxillary}\,\mathrm{sinuses}\,\mathrm{in}\,\mathrm{each}\,\mathrm{class}}$$

Model performance was also evaluated according to the classification for guidance regarding additional imaging or surgery; cyst or tumor versus healthy, cyst or tumor versus retention pseudocyst, and cyst or tumor versus healthy and retention pseudocyst. All experiments were conducted on Ubuntu 18.04 with Keras (version 2.4.3) and TensorFlow (version 2.4.1) frameworks using an NVIDIA TITAN Xp graphics card.

## Results

**Figure 2:**
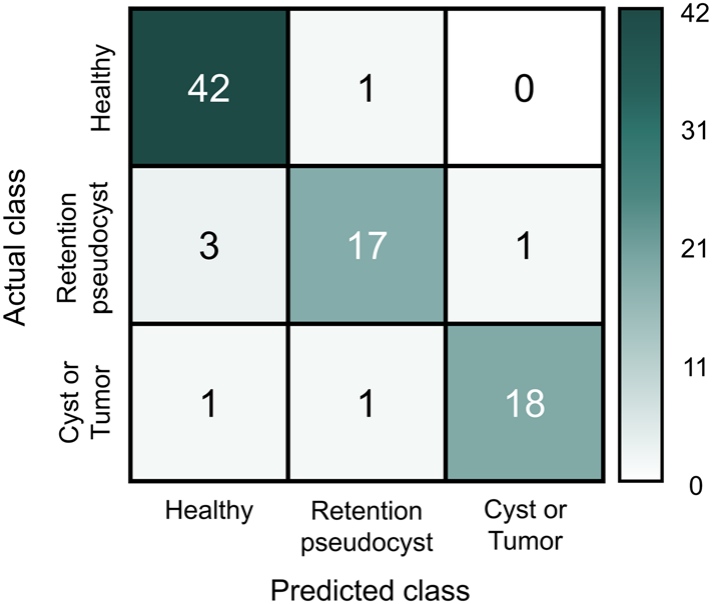
Classification matrix of the test dataset.

Table 2 shows the detection accuracies of the proposed model. The detection accuracies for class 0, class 1, and class 2 were 98%, 81%, and 90%, respectively, yielding a total accuracy of 92%. A classification matrix of the model using the test dataset is shown in Fig. 2.

**Table 2:** Performance of the model for maxillary sinus classification.

Group	Detection accuracy (95% CI)
Healthy (class 0)	98% (93.2–100%)
Retention pseudocyst (class 1)	81% (64.2–97.7%)
Cyst or tumor (class 2)	90% (76.9–100%)
Total sinuses (class 0, 1, and 2)	92% (85.8–97.6%)

*CI* Confidence interval.

**Figure 3:**
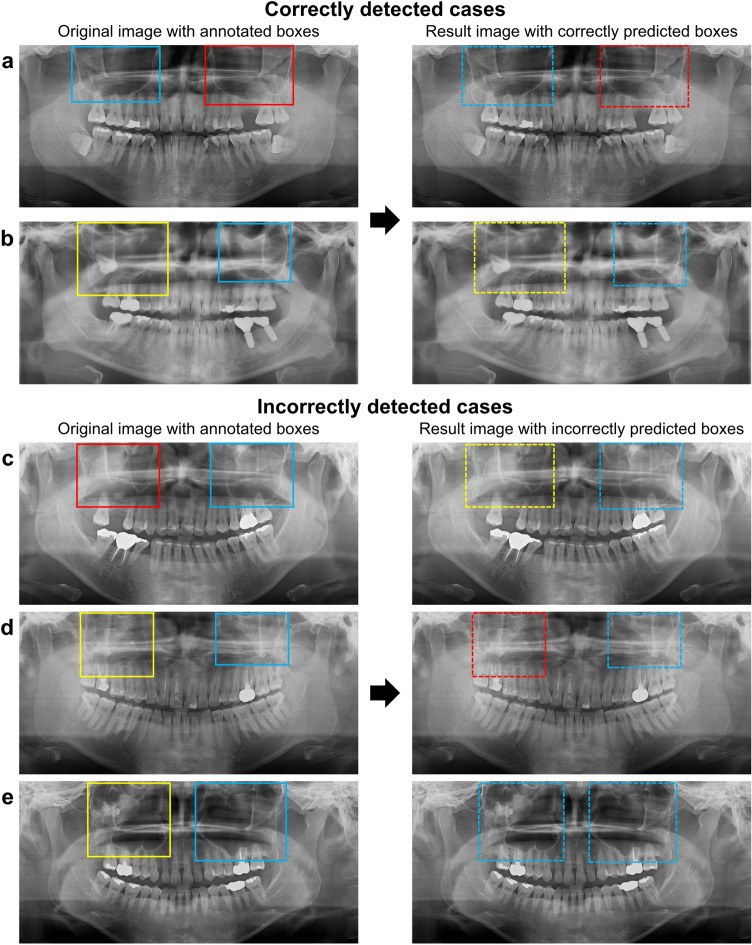
Examples of sinuses classified by the model. The left side of each case is the original panoramic radiograph with annotated boxes (solid line) and the right side of each case is the resulting panoramic radiograph with predicted boxes (dotted line) in a different color for each class (healthy: blue, retention pseudocyst: red, cyst or tumor: yellow). Examples of sinuses detected by the model with correct classes (**a**,**b**). Examples of sinuses detected by the model with incorrect classes (**c**–**e**). In the right maxillary sinuses, a retention pseudocyst was incorrectly predicted as a cyst or tumor (**c**), a cyst or tumor was incorrectly predicted as a retention pseudocyst (**d**), and a cyst or tumor was incorrectly predicted as healthy (**e**).

Table 3 presents the diagnostic performance of the model according to the classification for guidance of additional imaging or surgery. The classifications accuracies between the cyst or tumor group and the healthy group and between the cyst or tumor group and the retention pseudocyst group showed 98% and 95%, respectively. With reclassification according to the need for treatment, the model, which classified into the cyst or tumor group and the remaining two groups (healthy and retention pseudocyst), yielded an accuracy of 96%, a sensitivity of 90%, and a specificity of 98%. Figure 3 shows examples of sinuses automatically classified by the EfficientDet model.

**Table 3:** Performance of the model for guidance regarding additional imaging or surgery.

	Accuracy (%)	Sensitivity (%)	Specificity (%)
Cyst or tumor versus healthy	98	95	100
Cyst or tumor versus retention pseudocyst	95	95	94
Cyst or tumor versus healthy and retention pseudocyst	96	90	98

## Discussion

The present study proposed a CNN model to automatically classify retention pseudocysts in the maxillary sinuses on panoramic radiographs. The proposed model achieved a detection accuracy of 81% for the diagnosis of retention pseudocysts. For the detection and diagnosis of the sinuses using three classes (healthy, retention pseudocyst, and cyst or tumor), the proposed model achieved a high total diagnostic accuracy (92%).

Panoramic radiography is widely used as a screening modality in routine dental practice and is an essential tool for evaluating and diagnosing the maxillary sinuses^8,22^. However, due to imaging limitations such as overlapping anatomical features, blurring, and distortion, the diagnosis of sinus disease using panoramic imaging is sometimes limited even for experienced dental radiologists^23^.

Recent studies have developed deep learning models as diagnostic aids based on medical^24–26^ and dental images^11,27,28^. In the dental field, deep learning algorithms are commonly applied to panoramic radiographs^9,11,29^, and studies have attempted to make automatic diagnoses of sinuses on panoramic radiographs^12–14^. Kuwana et al.^12^ proposed a deep learning model using DetectNet, which was developed to detect the healthy sinuses, inflammatory sinuses, and sinuses with cysts or tumors, and reported reliable accuracy (98–100%). However, they classified retention pseudocyst as cyst, and the accuracy of the healthy group versus the all-lesion group (inflammatory sinuses and sinuses with cysts or tumors) was 89%. In clinical situations, it is important to distinguish between retention pseudocysts, which do not require treatment, and cysts or tumors that require surgery. Retention pseudocysts are occasionally misdiagnosed as cysts or tumors on panoramic radiographs, prompting additional CT scans or referral to a university hospital. In other previous studies^13,14^, models were developed to differentiate between healthy and inflamed sinuses (sinusitis) with clinical symptoms using the DetectNet and AlexNet algorithms, respectively, and the accuracy was 87.5%^13^ and 88.8%^14^. Inflammation of the sinus manifests as varying degrees of mucosal thickening, but the diagnosis of sinusitis depends on clinical manifestations rather than imaging. Therefore, this study attempted to develop a deep learning model based on the EfficientDet algorithm with a new classification of sinuses (healthy, retention pseudocyst, and cyst or tumor), considering that the differential diagnosis of retention pseudocyst is important. We also evaluated the accuracy of the model for distinguishing between the cyst or tumor group and the healthy group, as well as between the cyst or tumor group and the retention pseudocyst group, to address the question of whether additional treatment would be needed. The accuracy of discriminating the cyst or tumor group and the healthy group was 98%, similar to existing research (97–100%)^12^, and the accuracy of distinguishing the cyst or tumor group and retention pseudocyst group was 95%. Therefore, we expect that this model will be useful for the screening of sinuses in real dental clinics and will avoid unnecessary treatment and costs for patients.

Our study applied the EfficientDet algorithm to develop a model for diagnosing the maxillary sinuses. The EfficientDet algorithm, which is an object detection algorithm, is rarely used in dentistry, but it has shown excellent performance in the medical field^17–19^. Nawaz et al.^17^ obtained high performance with 97.9% accuracy in detecting glaucoma in fundus images using an EfficientDet algorithm, and Du et al.^19^ developed an EfficientDet model for discriminating breast cancer and achieved 92.6% accuracy. In a recent study^30^, the three models for detecting mesiodens on periapical radiography were developed using YOLOv3, RetinaNet and EfficientDet algorithms, and among these models, the EfficientDet model achieved the highest performance. The diagnostic performance of our model was also high, confirming the applicability of EfficientDet to panoramic radiographs.

This study has limitations in that it was conducted with a small dataset of 426 maxillary sinuses and all panoramic radiographs were acquired at a single institution. Also, the cases of sinuses with various degrees of mucosal thickening greater than 4 mm were not included in study. Further research using more images of sinuses with various conditions from multiple centers would enhance the clinical usefulness of the model.

## Conclusion

We proposed a deep learning model for automatically diagnosing retention pseudocysts in the maxillary sinuses on panoramic radiographs using the EfficientDet algorithm. The model obtained reliable accuracy and could be used to automatically diagnose the maxillary sinuses.

## Data Availability

The data generated and analyzed during the current study are not publicly available due to privacy laws and policies in Korea, but are available from the corresponding author on reasonable request.
